# 20-HETE mediates Ang II-induced cardiac hypertrophy via ROS and Ca^2+^ signaling in H9c2 cells

**DOI:** 10.1038/s41598-025-85992-2

**Published:** 2025-01-17

**Authors:** Jingyi Han, Jiaojiao Li, Lianlian Liu, Kaiyuan Li, Chun Zhang, Yong Han

**Affiliations:** Department of Physiology, Zunyi Medical University, Campus No.1 Road, Xinpu New District, Zunyi, 563006 Guizhou China

**Keywords:** 20-Hydroxyeicosatetraenoic acid, Angiotensin II, G protein-coupled receptor 75, Cardiac hypertrophy, Reactive oxygen species, Mechanisms of disease, Cardiac hypertrophy

## Abstract

In the vascular system, angiotensin II (Ang II) mediated vasoconstriction by inducing the production of 20-hydroxyeicosatetraenoic acid (20-HETE). However, the role of 20-HETE in Ang II-induced cardiac dysfunction had yet to be fully elucidated. This study investigated the effects of Ang II on CYP4A expression and 20-HETE production in H9c2 cells using RT-qPCR, Western blot, and ELISA. The role of 20-HETE in Ang II-induced cardiac hypertrophy was examined using DHE, MitoSOX, and JC-1 staining to evaluate reactive oxygen species (ROS) generation and mitochondrial membrane potential changes. The ERK/Akt and CaN/NFAT3 signaling pathways were analyzed through Western blot. Ang II was found to promote CYP4A expression and 20-HETE production in H9c2 cells via an AT1 receptor-dependent mechanism. Additionally, the upregulation of AT1 receptor expression by 20-HETE further confirms its facilitatory effect on the Ang II signaling pathway. Inhibition of 20-HETE synthesis or blockade of its receptor, G-protein-coupled receptor 75 (GPR75), significantly reversed Ang II-induced cardiac hypertrophy. This reversal was closely associated with 20-HETE-induced ROS production, oxidative stress, and activation of the Ca^2+^/CaN/NFAT3 signaling pathway. This study demonstrated that 20-HETE mediated Ang II-induced cardiac hypertrophy and, for the first time, highlighted the significant role of the GPR75 receptor in this process. These findings suggested that targeting 20-HETE reduction or blocking its receptor action could offer a novel therapeutic approach for cardiovascular diseases associated with Ang II.

**Supplementary Information:**

The online version contains supplementary material available at 10.1038/s41598-025-85992-2.

## Introduction

Pathological cardiac hypertrophy is a maladaptive remodeling process characterized by fetal gene activation, increased protein synthesis, cardiomyocyte enlargement, and interstitial fibrosis^1^. Persistent, uncontrolled hypertrophy leads to systolic and diastolic cardiac dysfunction and is recognized as an independent risk factor for cardiovascular diseases (CVDs), including arrhythmia and myocardial infarction^2^. It also provides a crucial structural basis for the eventual development of heart failure and sudden cardiac death^3^. However, pharmacological interventions capable of reversing this adverse process and restoring cardiac function remain limited.

The renin-angiotensin-aldosterone system (RAAS), a neurohumoral endocrine system, regulates cardiac function and plays a critical role in the development of pathological cardiac hypertrophy^4,5^. As a major effector molecule of the RAAS, angiotensin II (Ang II) triggers pathological hypertrophy by stimulating cardiomyocyte growth and interstitial fibrosis^5,6^. Clinical applications of angiotensin-converting enzyme inhibitors (ACEIs) and Ang II receptor blockers (ARBs) demonstrate alleviation of hypertrophy-related myocardial remodeling and are considered first-line treatments for hypertension and heart failure in CVDs^7^. However, targeting the classic RAAS members as therapeutic options has limitations. For instance, cardiac chymase can activate angiotensinogen independently of the angiotensin-converting enzyme (ACE) pathway, bypassing ACEI effects and releasing Ang II in an autocrine manner. Ang II produced via this alternative pathway exerts effects without binding to membrane receptors, rendering it resistant to ARBs^8^. Additionally, these drugs cannot fully prevent chronic heart failure progression and associated complications. Therefore, novel therapeutic targets are needed to regulate Ang II-related myocardial remodeling and mitigate the progression of CVDs.

20-Hydroxyeicosatetraenoic acid (20-HETE), a product of arachidonic acid (AA) ω-hydroxylation by the cytochrome P450 4 A/4F (CYP4A/4F) enzyme system^9–11^, is a potent vasoconstrictor that induces vascular smooth muscle cell (VSMC) contraction, migration, and proliferation. It also contributes to endothelial cell dysfunction and inflammation, facilitating CVDs such as hypertension and stroke^12,13^. In ischemic heart diseases, including myocardial ischemia-reperfusion injury (MIRI), various stimuli elevate 20-HETE production^14,15^. Subsequently, 20-HETE exacerbates myocardial injury by promoting oxidative stress, inducing Ca^2+^ overload, and causing mitochondrial dysfunction^16–18^. Furthermore, 20-HETE is implicated in cardiac hypertrophy induced by multiple stimuli. In cardiac hypertrophy models triggered by isoproterenol (ISO) and aryl hydrocarbon receptor (AhR) activation, increased CYP4A/4F enzyme expression elevates 20-HETE levels, and inhibiting its synthesis significantly mitigates hypertrophy^19,20^. However, the underlying mechanisms require further elucidation.

Previous studies confirm that Ang II in the vascular system promotes the AA metabolic pathway and stimulates 20-HETE production, contributing to Ang II-induced vasoconstriction and hypertension^21–24^. This highlights the critical role of 20-HETE in Ang II-mediated blood pressure regulation and vascular tone modulation. However, its interaction with Ang II within the heart remains largely unexplored. Our previous research found that Ang II increases CYP4A expression and 20-HETE production in neonatal rat cardiomyocytes via the angiotensin II type 1 (AT1) receptor, and that inhibiting 20-HETE synthesis effectively reverses Ang II-induced cardiomyocyte apoptosis^25^. Nevertheless, the involvement of 20-HETE in Ang II-induced cardiac hypertrophy and its mechanisms remains unclear. Therefore, this study explores the impact of inhibiting 20-HETE synthesis on Ang II-induced myocardial hypertrophy in H9c2 cardiomyocytes, focusing on the roles of reactive oxygen species (ROS), Ca^2+^-mediated signaling pathways, and the recently identified 20-HETE receptor, G-protein-coupled receptor 75 (GPR75).

## Materials and methods

### Reagents

Ang II, losartan, and PD123319 were purchased from Sigma-Aldrich (St. Louis, MO, USA). Setanaxib was obtained from MedChemExpress (Monmouth Junction, NJ, USA). 20-HETE, HET0016, and AAA were purchased from Cayman Chemical (Ann Arbor, MI, USA). The 20-HETE ELISA Kit was sourced from Detroit R&D (Detroit, MI, USA). The DHE Staining Kit and BCA Protein Assay Kit were obtained from Solarbio (Beijing, China). Minute™ Cytoplasmic and Nuclear Extraction Kit was purchased from Invent Biotechnologies (Beijing, China). The JC-1 Assay Kit, Mn-SOD Assay Kit, and Fluo-4 AM dye were obtained from Beyotime (Shanghai, China). The MitoSOX Red Mitochondrial Superoxide Indicator was acquired from YEASEN (Shanghai, China). The NADPH Activity Quantification Kit was obtained from Genmed (Shanghai, China). The Prime Script RT reagent Kit and TB Green Premix Ex Taq were sourced from Takara Bio (Dalian, China). RNA synthesis reagents were provided by GeneRay (Shanghai, China).

### Cell culture and treatments

The H9c2 cardiac cell line was obtained from the Cell Bank/Stem Cell Bank (Chinese Academy of Sciences, Shanghai, China) and cultured in Dulbecco’s Modified Eagle Medium (DMEM; Gibco, USA) supplemented with 10% fetal bovine serum (FBS; MRC, Jiangsu, China) and 1% penicillin-streptomycin mixture (Solarbio, China) at 37 °C in a humidified incubator with 5% CO_2_. Cells were fed every 2 to 3 days and used for experiments at 70–80% confluence. Following overnight serum starvation, H9c2 cells were stimulated for 24 h with Ang II (1 µmol/L)^26^ or 20-HETE (100 nmol/L)^27^ to induce hypertrophy. Pretreatments with HET0016 (10 µmol/L)^16,17^, AAA (10 µmol/L)^28^, losartan (1 µmol/L)^29^, PD123319 (1 µmol/L), or setanaxib (10 µmol/L)^30^ were administered one hour prior to Ang II or 20-HETE treatments, as dictated by the experimental design. After treatments, cells were harvested for subsequent analyses.

### Measurement of cell surface area and protein content

To measure cell surface area, H9c2 cells were fixed in 4% paraformaldehyde for 1–2 min at room temperature, stained with 0.5% ammonium oxalate crystal violet solution (Solarbio, China) for 15 min, and rinsed with double-distilled water. Cell morphology was observed under a light microscope, and cell area was quantified using ImageJ 1.48 software (NIH, MD, USA).

For total protein extraction from H9c2 cells, drug-treated cells were harvested, washed with precooled phosphate-buffered saline (PBS), and lysed in radioimmunoprecipitation assay (RIPA) buffer (Solarbio, China). The lysates were incubated on ice for 30 min and then centrifuged at 14,000 g for 7 min at 4 °C. The supernatant containing the total protein extract was collected. Protein concentration was subsequently measured using the bicinchoninic acid (BCA) protein assay kit (Solarbio, China).

### Measurement of ROS generation

Intracellular ROS production in H9c2 cells was assessed using dihydroethidium (DHE; Beyotime, China) staining. After treatments, cells were washed with DMEM, incubated with 10 µmol/L DHE at 37 °C for 40 min in the dark, and rinsed with PBS to remove excess dye. Fluorescence detection was performed using a confocal laser scanning microscope (Leica, Germany) with excitation and emission wavelengths of 535 nm and 610 nm, respectively. Fluorescence intensities were quantified using LAS AF Lite 2.6 software (Leica, Germany).

### Measurement of NADPH oxidase and Mn-SOD activities

NADPH oxidase activity was evaluated by measuring NADPH-dependent superoxide production using the SOD-inhibitable cytochrome c reduction method, as previously described^18^. H9c2 cells were lysed to extract total protein (1 mg/mL) and aliquoted into a 96-well plate. Samples were incubated with cytochrome c (500 µmol/L) and NADPH (100 µmol/L) for 30 min, with or without superoxide dismutase (SOD; 200 U/mL). Cytochrome c reduction was measured at 550 nm using a microplate reader (Bio-Rad, USA). Superoxide production was calculated as the difference in absorbance with and without SOD to determine NADPH oxidase activity.

Mn-SOD activity was assessed using the WST-8 colorimetric assay with the Cu/Zn-SOD and Mn-SOD Assay Kit (Beyotime, China). Total protein in H9c2 cells was quantified using the BCA protein assay kit (Solarbio, China), as described above. Cu/Zn-SOD inhibitors were added to the samples, followed by the WST-8/enzyme working solution, and incubated for 30 min at 37 °C. Absorbance was measured at 450 nm using a microplate reader (Bio-Rad, USA), and Mn-SOD activity was calculated according to the manufacturer’s protocol.

### Detection of mitochondrial ROS (Mito-ROS)

Mito-ROS production in H9c2 cells was evaluated using the MitoSOX Red indicator (YEASEN, China), following the manufacturer’s instructions. After incubation with 5 µmol/L MitoSOX Red in the dark for 10 min at 37 °C and rinsing with DMEM, cells were stained with 10 µmol/L 4′,6-diamidino-2-phenylindole (DAPI) for 5 min to visualize nuclei. Images were captured using a confocal laser scanning microscope (Leica, Germany) with excitation at 510 nm and emission at 580 nm. Quantification of fluorescence intensities was performed using LAS AF Lite software.

### Immunofluorescence analysis of 8-hydroxy-2’-deoxyguanosine (8-OhdG)

For immunofluorescence analysis, H9c2 cells were fixed with 4% formaldehyde in PBS for 15 min at room temperature and permeabilized with 0.1% Triton X-100 (Beyotime, China) for 10 min. Following blocking with 5% normal goat serum for 1 h, cells were incubated with a primary antibody against 8-OHdG (Abcam, diluted 1:1000) overnight at 4 °C. The next day, cells were incubated with a FITC-labeled goat anti-rabbit IgG secondary antibody (Santa Cruz, diluted 1:300) for 1 h at room temperature, followed by DAPI staining for 5 min to visualize nuclei. Images were captured using a fluorescence microscope (Olympus, Japan), and fluorescence intensities were quantified using ImageJ software.

### **Detection of mitochondrial membrane potential (*ΔΨm*)**

*ΔΨm* was detected using 5,5’,6,6’-tetrachloro-1,1’,3,3’-tetramethylbenzimidazolylcarbocyanine iodide (JC-1; Solarbio, China). H9c2 cells were incubated with 10 µg/mL JC-1 in the dark for 30 min at 37 °C. After washing with PBS, fluorescence observations were made using a confocal laser scanning microscope (Leica, Germany). Red and green fluorescence were captured with excitation at 585 nm and 514 nm and emission at 590 nm and 529 nm, respectively. Fluorescence intensities were quantified using LAS AF Lite 2.6 software.

### Measurement of intracellular Ca^2+^ level

Cytosolic Ca^2+^ levels were assessed using the cell-permeable Ca^2+^ fluorescent probe Fluo-4 AM (Beyotime, China). H9c2 cells were incubated with 5 µmol/L Fluo-4 AM and 0.04% Pluronic F-127 (Solarbio, China) at 37 °C in the dark for 50 min. After washing, cells were further incubated for an additional 20 min to allow the conversion of Fluo-4 AM to Fluo-4. Fluorescence intensity was measured using a fluorescence microscope (Olympus, Japan) with an excitation wavelength of 488 nm. ImageJ software was used for analysis.

### Measurement of 20-HETE level

The 20-HETE level in H9c2 cells was determined by enzyme immunoassay using a 20-HETE ELISA Kit (Detroit R&D, USA). Following drug treatments, cells were harvested and sonicated. 20-HETE was extracted using ethyl acetate and dissolved in DMSO. Samples were loaded onto a 96-well plate coated with anti-20-HETE antibody, and the assay was performed according to the manufacturer’s instructions. The plate was read at 450 nm using a microplate reader (Bio-Rad, USA), and 20-HETE levels were calculated from the standard curve.

### Reverse transcription-quantitative polymerase chain reaction (RT-qPCR)

Total RNA was extracted from H9c2 cells using RNAiso Plus reagent (Takara, China), and cDNA synthesis was performed using the Prime Script RT reagent kit (Takara, China), following the manufacturer’s protocols. RT-qPCR was conducted on the QuantStudio 6 Flex Real-Time PCR System (Thermo Fisher Scientific, USA) using TB Green Premix Ex Taq (Takara, China). Reaction conditions were as follows: initial denaturation at 94 °C for 30 s, followed by 40 cycles of 94 °C for 5 s and 60 °C for 30 s. Relative mRNA expression was determined using the 2^−ΔΔCt^ method, with GAPDH as the endogenous control. The primers used in this study are detailed in Table 1.

**Table 1:** Primer sequences of RT-qPCR.

Gene	Forward Primers 5’−3’	Reverse Primers 5’−3’
AT1	CCCACTCAAGCCTGTCTACGAA	GTGTGCTTTGAACCTGTCACTCC
CYP4A1	TTGAGCTACTGCCAGATCCCAC	CCCATTTTTGGACTTCAGCACA
ANP	CGGACAAAGGCTGAGAGAGAAAC	AAAAGGCCAGGAAGAGGAAGAAG
BNP	GACAAGAGAGAGCAGGACACCAT	TAAGGAAAAGCAGGAGCAGAATCAT
GADPH	TCTCTGCTCCTCCCTGTTC	ACACCGACCTTCACCATCT

### Western blot analysis

Total protein extraction was performed as described in the “Measurement of Cell Surface Area and Protein Content” section. Cytoplasmic and nuclear proteins were extracted using the Cytoplasmic and Nuclear Extraction Kit. After drug treatment, H9c2 cells were washed with prechilled PBS, scraped, and incubated on ice with 50 µL of Cytoplasmic Extraction Buffer for 10 min. The mixture was then centrifuged at 14,000 g for 10 min at 4 °C, and the supernatant containing cytoplasmic proteins was collected. The pellet was washed twice with PBS, centrifuged again at 14,000 g for 5 min at 4 °C, and PBS was aspirated. Nuclear proteins were extracted by adding 30 µL of Nuclear Extraction Buffer to the pellet, followed by incubation on ice for 5 min and centrifugation at 15,000 g for 1 min at 4 °C. The resulting supernatant represented the nuclear protein fraction.

For immunoblot analysis, proteins were separated by electrophoresis on a 10% SDS-PAGE gel and transferred onto a PVDF membrane (Millipore, USA). Membranes were blocked with 5% BSA in tris-buffered saline with Tween-20 (TBS-T) for 2 h, followed by overnight incubation at 4 °C with primary antibodies: anti-AT1 (Proteintech, 1:500), anti-CYP4A (Santa Cruz, 1:500), anti-NOX2 (Invitrogen, 1:2000), anti-NOX4 (Invitrogen, 1:2000), anti-ERK1/2 (Abcam, 1:10,000), anti-Akt (Abcam, 1:10,000), anti-p47phox (Santa Cruz, 1:1000), anti-p-ERK1/2 (CST, 1:2000), anti-p-Akt (CST, 1:2000), anti-p-p47phox (Santa Cruz, 1:1000), anti-CaN (CST, 1:800), anti-NFAT3 (CST, 1:800), and anti-β-actin (Proteintech, 1:5000). The next day, membranes were washed three times with TBS-T and incubated with HRP-conjugated secondary antibodies for 1 h. Protein expression levels were normalized to β-actin as the loading control. Bands were visualized using an ECL system, and intensities were quantified using ImageJ software.

#### Statistical analysis

All data were presented as mean ± SD. Statistical analyses were conducted using SPSS version 29.0 software (IBM, USA). One-way analysis of variance (ANOVA) was used to evaluate significant differences among multiple groups, followed by post hoc group comparisons using the Least Significant Difference (LSD) test. A value of *P* < 0.05 was considered statistically significant.

## Results

### Inhibition of 20-HETE production or blockade of its action prevents Ang II-induced H9c2 cells hypertrophy

**Fig. 1:**
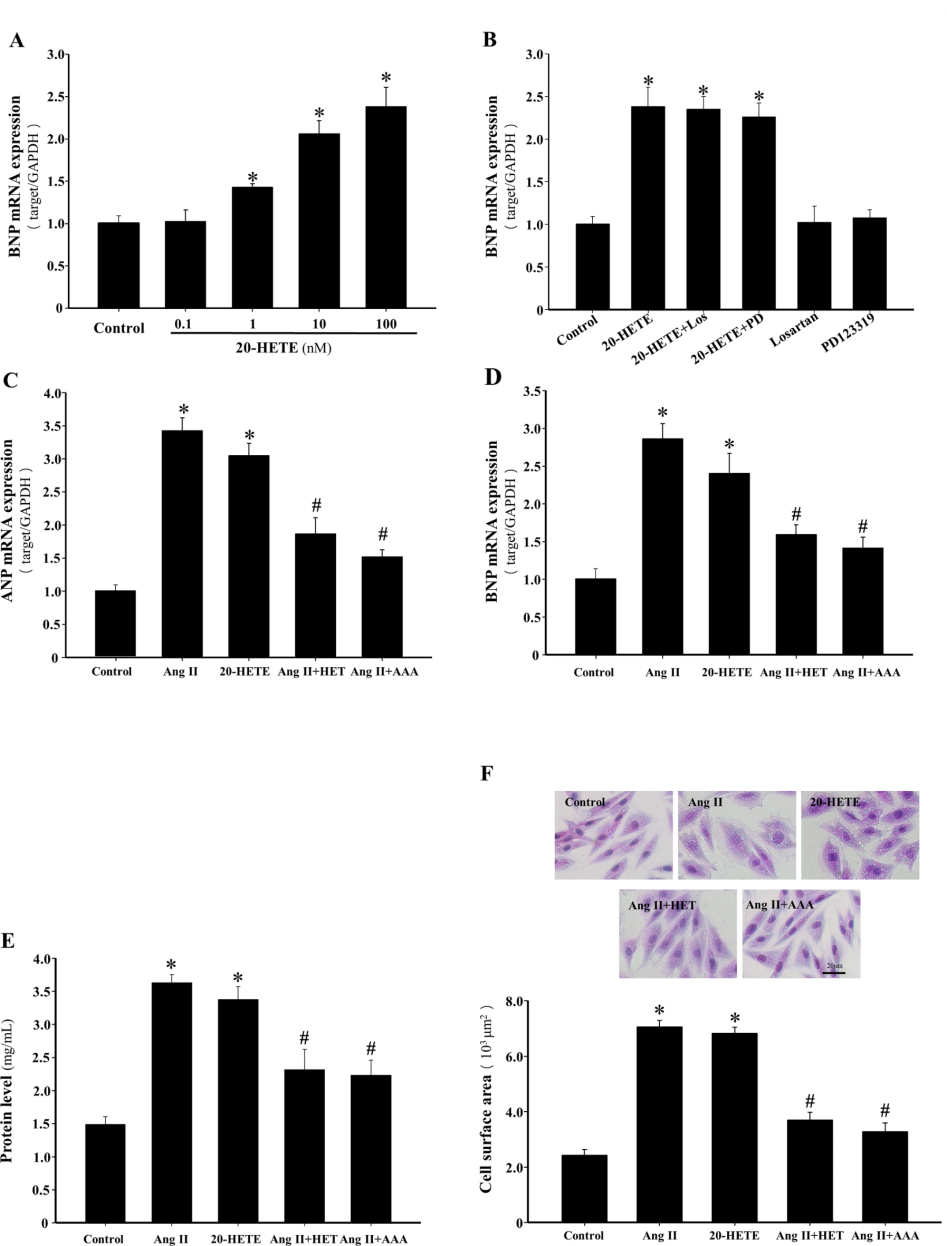
Inhibition of 20-HETE production or blockade of its action prevents Ang II-induced H9c2 cells hypertrophy. (**A**) H9c2 cells were treated with different doses of 20-HETE (0.1–100 nmol/L) for 24 h, and BNP mRNA levels were assessed by RT-qPCR analysis. *P < 0.05 vs. Control group, *n* = 5. (**B**) RT-qPCR analysis of BNP mRNA levels in the indicated groups. *P < 0.05 vs. Control group, *n* = 5. (**C**–**D**) The ANP and BNP mRNA expression levels were measured by RT-qPCR, and the expression levels in the different groups were quantified. *P < 0.05 vs. Control group, ^#^*P* < 0.05 vs. Ang II or 20-HETE group, *n* = 5. (**E**) BCA method for total protein content. *P < 0.05 vs. Control group, ^#^*P* < 0.05 vs. Ang II or 20-HETE group, *n* = 5. (**F**) Representative images of crystal violet stained H9c2 cells (scale bar: 20 μm) and the quantification of cell surface area after different treatments. *P < 0.05 vs. Control group, ^#^*P* < 0.05 vs. Ang II or 20-HETE group, *n* = 3.

Treatment of H9c2 cells with 20-HETE (0.1–100 nmol/L) for 24 h resulted in a dose-dependent increase in brain natriuretic peptide (BNP) mRNA levels (*P* < 0.05, Fig. 1A). To assess the impact of Ang II on 20-HETE-induced hypertrophy, H9c2 cells were pretreated with losartan (1 µmol/L) or PD123319 (1 µmol/L) for 1 h before 20-HETE exposure. Pretreatment with these antagonists did not significantly affect the hypertrophic response to 20-HETE (*P* > 0.05, Fig. 1B), indicating that 20-HETE-induced hypertrophy was independent of Ang II signaling. However, pretreatment with HET0016 (10 µmol/L), an inhibitor of 20-HETE synthase, or the 20-HETE receptor antagonist N-disodium succinate-20-hydroxyeicosa-6(Z),15(Z)-diencarboxamide (AAA) (10 µmol/L) significantly reduced Ang II-induced atrial natriuretic peptide (ANP) and BNP mRNA expression (*P* < 0.05, Fig. 1C and D), as well as Ang II-induced increases in total protein content and cell surface area (*P* < 0.05, Fig. 1E and F). Additionally, 20-HETE (100 nmol/L) treatment for 24 h induced hypertrophic effects in H9c2 cells, evidenced by the expression of fetal-type genes (ANP and BNP), increased protein synthesis, and cell enlargement (*P* < 0.05, Fig. 1A–F), mimicking the hypertrophic actions of Ang II.

### Ang II stimulates 20-HETE production and enhances CYP4A1 expression in H9c2 cells

**Fig. 2:**
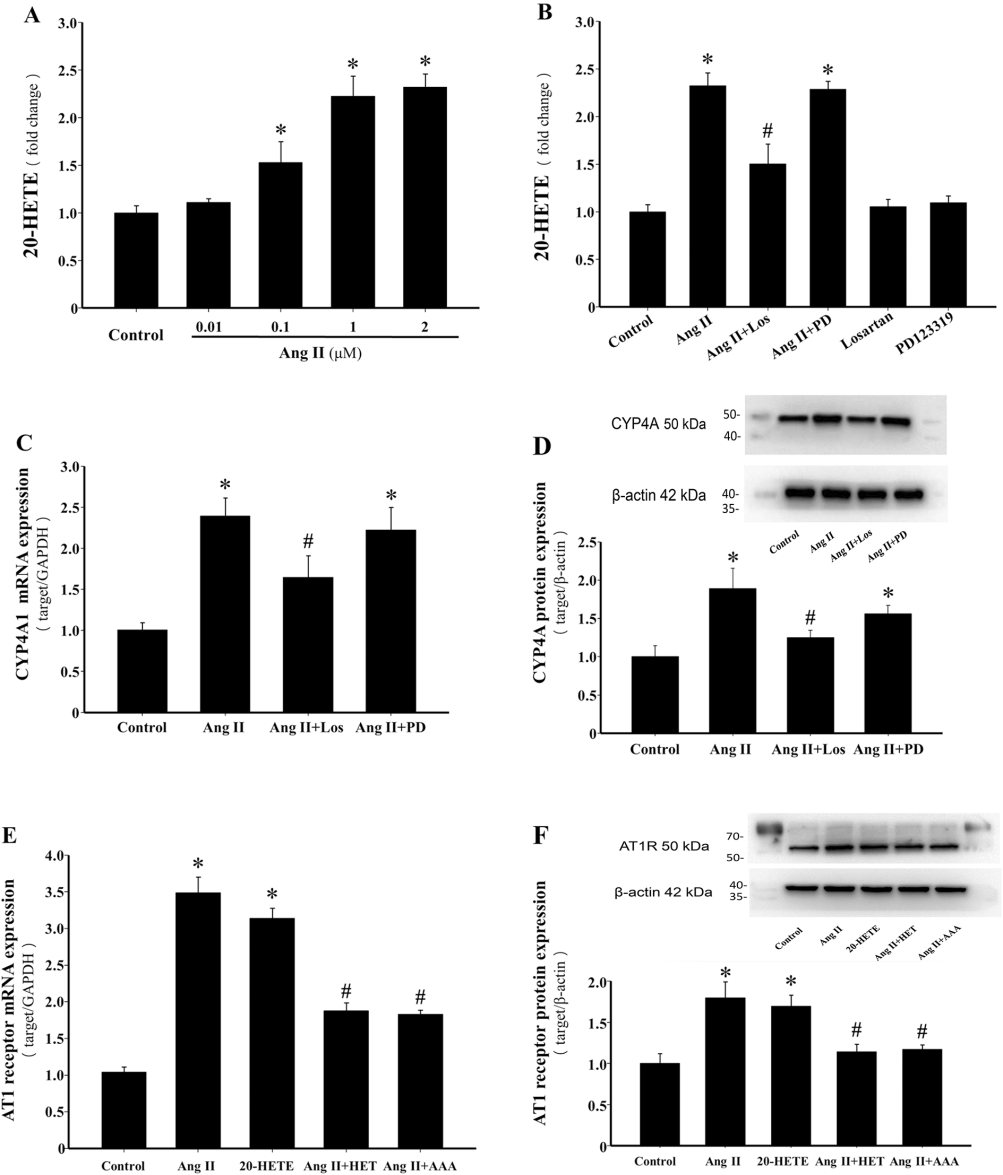
Ang II stimulates 20-HETE production and enhances CYP4A1 expression in H9c2 cells. (**A**) ELISA was used to measure 20-HETE content in H9c2 cells treated with varying doses of Ang II (0.01–2 µmol/L) for 24 h. *P < 0.05 vs. Control group, *n* = 3. (**B**) Effects of Losartan and PD123319 pretreatment on the Ang II-induced 20-HETE production in H9c2 cells. *P < 0.05 vs. Control group, ^*#*^*P* < 0.05 vs. Ang II group, *n* = 3. (**C**) RT-qPCR analysis of the expression of CYP4A1 with different treatments. *P < 0.05 vs. Control group, ^*#*^*P* < 0.05 vs. Ang II group, *n* = 5. (**D**) Western blot analysis of the protein levels of CYP4A in H9c2 cells with different treatments. *P < 0.05 vs. Control group, ^*#*^*P* < 0.05 vs. Ang II group, *n* = 3. (**E**) AT1 receptor mRNA expression levels in the different groups were quantified. *P < 0.05 vs. Control group, ^*#*^*P* < 0.05 vs. Ang II or 20-HETE group, *n* = 3. (**F**) Representative blots and quantification of AT1 receptor protein expression. *P < 0.05 vs. Control group, ^*#*^*P* < 0.05 vs. Ang II or 20-HETE group, *n* = 3.

Exposure to Ang II (0.01–2 µmol/L) increased 20-HETE levels in a dose-dependent manner (*P* < 0.05), with a significant two-fold increase observed at 1 µmol/L (*P* < 0.05, Fig. 2A). To investigate the mechanisms underlying Ang II-induced 20-HETE production, H9c2 cells were pretreated with losartan or PD123319 for 1 h before Ang II (1 µmol/L) stimulation. Pretreatment with losartan, but not PD123319, significantly inhibited Ang II-induced 20-HETE synthesis (*P* < 0.05, Fig. 2B), indicating an AT1 receptor-mediated pathway. The CYP4A enzyme family, including CYP4A1, CYP4A2, and CYP4A3, is responsible for 20-HETE production in rats, with CYP4A1 being the most efficient isoform for arachidonic acid metabolism to 20-HETE^31^. Ang II significantly upregulated CYP4A1 mRNA expression, an effect inhibited by losartan but not PD123319 (*P* < 0.05, Fig. 2C). Western blot analysis confirmed increased CYP4A protein levels in response to Ang II treatment (*P* < 0.05, Fig. 2D).

Previous studies demonstrated that 20-HETE promotes ACE expression in the vascular system. To further evaluate this, the impact of 20-HETE on AT1 receptor expression in H9c2 cardiomyocytes was examined. As shown in Figs. 2E and F and 20-HETE significantly upregulated AT1 receptor mRNA and protein levels (*P* < 0.05). Furthermore, treatment with HET0016 or AAA markedly inhibited Ang II-induced AT1 receptor expression (*P* < 0.05, Fig. 2E and F), suggesting that Ang II-induced AT1 receptor upregulation is closely associated with its stimulation of 20-HETE production.

### Inhibition of 20-HETE production or blockade of its action attenuates Ang II-induced intracellular ROS accumulation in H9c2 cells

**Fig. 3:**
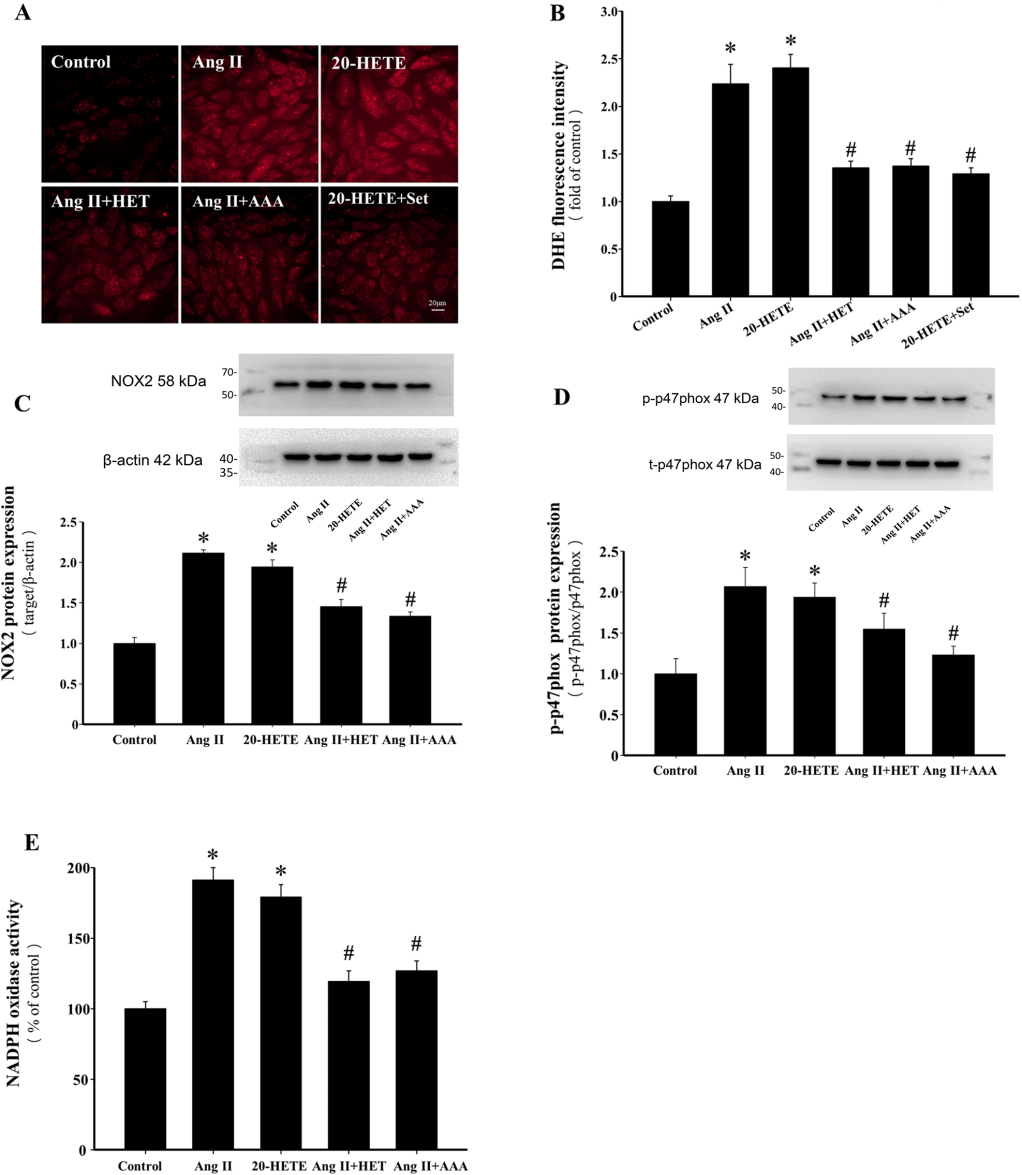
Inhibition of 20-HETE production or blockade of its action attenuates Ang II-induced intracellular ROS accumulation in H9c2 cells. (**A**) Representative images of the levels of ROS generation as measured by DHE staining (scale bar: 20 μm). (**B**) Analysis of mean fluorescence intensity in each group. *P < 0.05 vs. Control group, ^*#*^*P* < 0.05 vs. Ang II or 20-HETE group, *n* = 3. (**C**–**D**) Western blot analysis and quantification of the expression of NOX2 and p-p47phox in the indicated groups. *P < 0.05 vs. Control group, ^*#*^*P* < 0.05 vs. Ang II or 20-HETE group, *n* = 3. (**E**) NADPH oxidase activity was measured by cytochrome c reduction method. *P < 0.05 vs. Control group, ^*#*^*P* < 0.05 vs. Ang II or 20-HETE group, *n* = 5.

Intracellular ROS generation plays a pivotal role in Ang II-induced cardiac hypertrophy^32^. The effects of inhibiting 20-HETE production or blocking its action on Ang II-induced ROS generation were assessed. Pretreatment with HET0016 or AAA significantly reduced Ang II-induced ROS generation in H9c2 cells (*P* < 0.05, Fig. 3A and B). Similarly, treatment of H9c2 cells with 20-HETE promoted ROS generation, which was significantly attenuated by pretreatment with Setanaxib (GKT137831, 10 µmol/L), a specific NOX1/NOX4 inhibitor (*P* < 0.05, Fig. 3A and B). These results suggest that 20-HETE contributes to Ang II-induced ROS generation in H9c2 cells by activating NADPH oxidase. NOX2, an isoform of NADPH oxidase, plays a critical role in Ang II-induced ROS generation in cardiomyocytes and is closely associated with the phosphorylation of cytosolic p47phox^33^. Pretreatment with HET0016 or AAA significantly inhibited Ang II-induced NOX2 expression and p47phox phosphorylation (*P* < 0.05, Fig. 3C and D). Additionally, both HET0016 and AAA effectively reduced Ang II-induced NADPH oxidase activity (*P* < 0.05, Fig. 3E).

### Inhibition of 20-HETE production or blockade of its action reduces Ang II-induced mt-ROS generation and oxidative stress in mitochondria

**Fig. 4:**
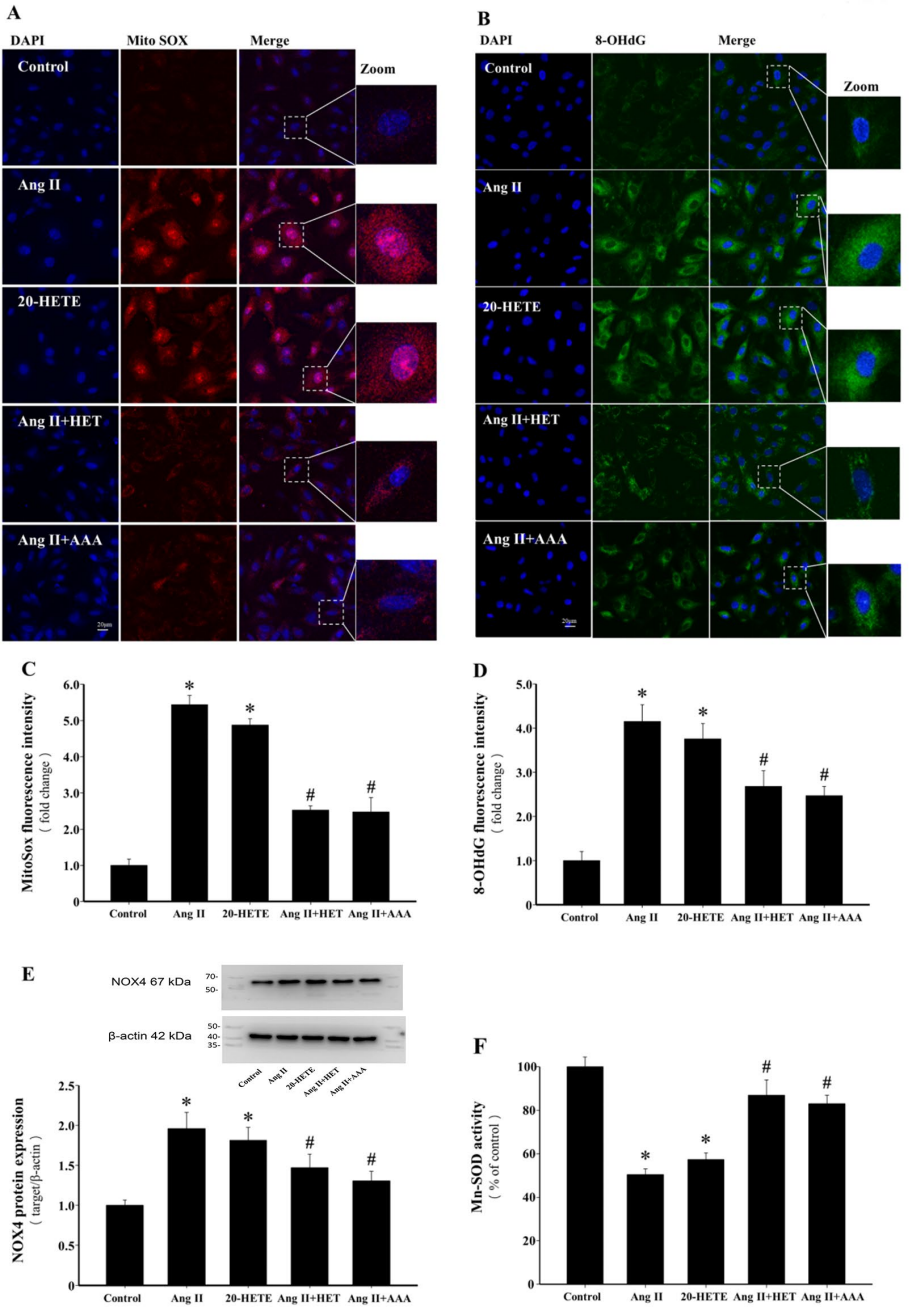
Inhibition of 20-HETE production or blockade of its action reduces Ang II-induced mt-ROS generation and oxidative stress in mitochondria. (**A**) Representative immunofluorescence images of MitoSOX Red (red) and DAPI (blue) staining (scale bar: 20 μm). (**B**) Representative images of 8-OHdG (green) and DAPI (blue) stained H9c2 cells. (scale bar: 20 μm). (**C**) Quantification of mean fluorescence intensity of mt-ROS generation in each group. *P < 0.05 vs. Control group, ^*#*^*P* < 0.05 vs. Ang II or 20-HETE group, *n* = 3. (**D**) Quantification of mean fluorescence intensity of 8-OHdG expression in each group. *P < 0.05 vs. Control group, ^*#*^*P* < 0.05 vs. Ang II or 20-HETE group, *n* = 3. (**E**) Representative blots and quantification of NOX4 protein expression. *P < 0.05 vs. Control group, ^*#*^*P* < 0.05 vs. Ang II or 20-HETE group, *n* = 3. (**F**) Activity of Mn-SOD was measured by WST-8 colorimetric assay. *P < 0.05 vs. Control group, ^*#*^*P* < 0.05 vs. Ang II or 20-HETE group, *n* = 5.

Mitochondria are both sources of ROS and targets for ROS-induced oxidative damage^32^. Treatment with Ang II and 20-HETE significantly increased mitochondrial ROS (mt-ROS) production, while pretreatment with HET0016 or AAA significantly suppressed Ang II-induced mt-ROS generation (*P* < 0.05, Fig. 4A and C). Immunofluorescence analysis of 8-OHdG, a biomarker of mitochondrial DNA (mt-DNA) damage, revealed that Ang II and 20-HETE treatment significantly increased 8-OHdG levels, an effect significantly reduced by HET0016 or AAA pretreatment (*P* < 0.05, Fig. 4B and D). NOX4, a NADPH oxidase isoform expressed in myocardial mitochondria, is a key contributor to Ang II-induced mt-ROS production^34^. Treatment with Ang II and 20-HETE significantly increased NOX4 expression (*P* < 0.05, Fig. 4E), while pretreatment with HET0016 or AAA effectively inhibited Ang II-induced NOX4 upregulation (*P* < 0.05). Furthermore, Ang II-induced oxidative stress was associated with impaired antioxidant capacity, as indicated by a reduction in Mn-SOD activity. Pretreatment with HET0016 or AAA significantly restored Mn-SOD activity (*P* < 0.05, Fig. 4F).

### **Inhibition of 20-HETE production or blockade of its action suppresses Ang II-induced mitochondrial membrane potential (*ΔΨm*) depolarization**

**Fig. 5:**
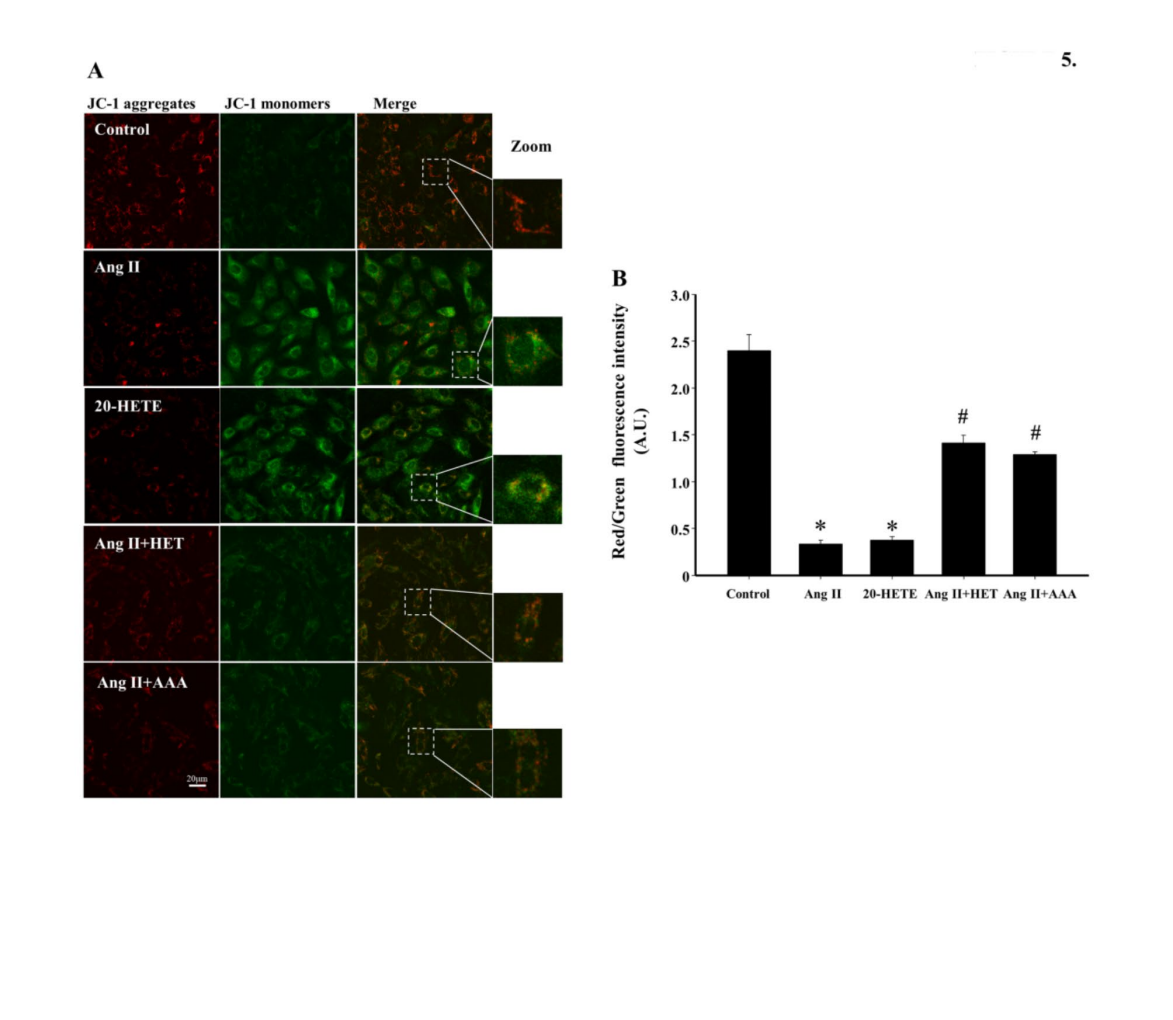
Inhibition of 20-HETE production or blockade of its action suppresses Ang II-induced *ΔΨm* depolarization. (**A**) Representative immunofluorescence images of JC-1 staining; red fluorescence indicates JC-1 aggregates, and green fluorescence indicates monomeric JC-1 (scale bar: 20 μm). (**B**) Quantification of red/green fluorescence intensity of *ΔΨm* levels in each group. *P < 0.05 vs. Control group, ^*#*^*P* < 0.05 vs. Ang II or 20-HETE group, *n* = 3.

Disruptions in *ΔΨm *plays a significant role in Ang II-induced cardiac hypertrophy^32^. The JC-1 fluorescence assay was used to evaluate the effects of inhibiting 20-HETE production or blocking its action on *ΔΨm* levels in H9c2 cells. Ang II and 20-HETE treatments significantly reduced *ΔΨm* levels compared to control group (*P* < 0.05, Fig. 5A and B). However, HET0016 or AAA pretreatment significantly reversed the Ang II-induced decline in *ΔΨm* (*P* < 0.05). These findings suggest that intracellular 20-HETE overproduction induced by Ang II contributes to ROS generation, mitochondrial damage, and cardiac hypertrophy.

### Inhibition of 20-HETE production or blockade of its action attenuates Ang II-induced phosphorylation of ERK1/2 and Akt

**Fig. 6:**
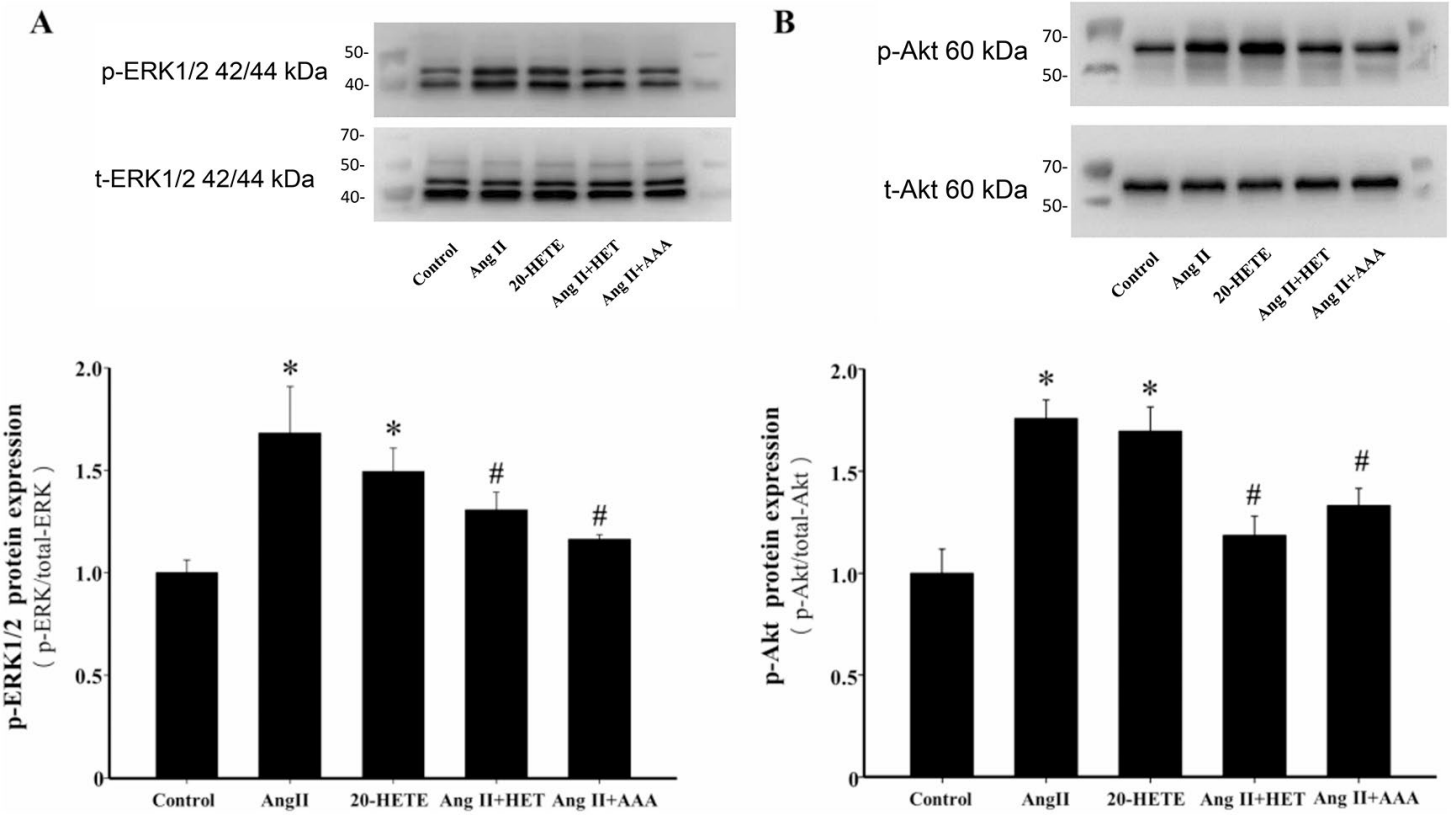
Inhibition of 20-HETE production or blockade of its action attenuates Ang II-induced phosphorylation of ERK1/2 and Akt in H9c2 cells. The total and phosphorylated levels of ERK1/2 and Akt proteins were detected and quantified. (**A**) Representative blots and quantification of p-ERK1/2 protein expression in the indicated groups. *P < 0.05 vs. Control group, ^*#*^*P* < 0.05 vs. Ang II or 20-HETE group, *n* = 3. (**B**) Representative blots and quantification of p-Akt in the indicated groups. *P < 0.05 vs. Control group, ^*#*^*P* < 0.05 vs. Ang II or 20-HETE group, *n* = 3.

ROS-mediated activation of the MAPK/ERK and PI3K/Akt signaling pathways is a critical mechanism in Ang II-induced cardiac hypertrophy^35^. Treatment of H9c2 cells with Ang II and 20-HETE significantly increased phosphorylation levels of ERK1/2 and Akt (*P* < 0.05, Fig. 6A and B). However, pretreatment with HET0016 or AAA significantly reversed these phosphorylation increases (*P* < 0.05, Fig. 6A and B). These results indicate that inhibiting 20-HETE production or blocking its action protects against Ang II-induced cardiac hypertrophy, primarily by suppressing ROS-mediated activation of MAPK/ERK and PI3K/Akt signaling pathways.

### Inhibition of 20-HETE production or blockade of its action alleviates intracellular Ca^2+^ overload and Nuclear Factor of Activated T cells 3 (NFAT3) activation induced by Ang II

**Fig. 7:**
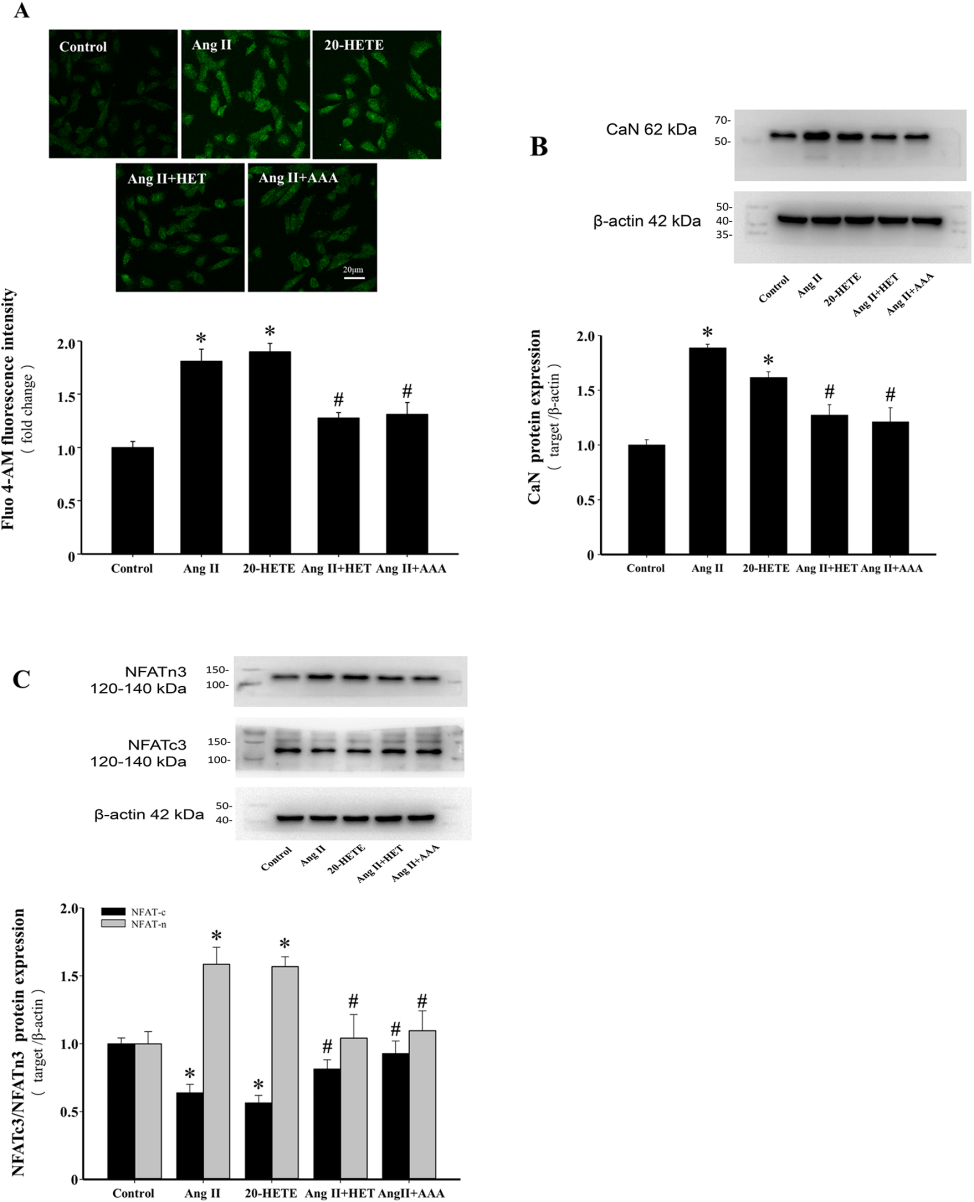
Inhibition of 20-HETE production or blockade of its action alleviates intracellular Ca^2+^ overload and NFAT3 activation induced by Ang II. (**A**) Representative immunofluorescence images of Fluo-4 AM (scale bar: 20 μm) and quantification of mean fluorescence intensity of intracellular Ca^2+^ concentrations in the different groups. *P < 0.05 vs. Control group, ^*#*^*P* < 0.05 vs. Ang II or 20-HETE group, *n* = 4. (**B**–**C**) Relative protein expression levels of CaN, NFATc3 and NFATn3 in the indicated groups. *P < 0.05 vs. Control group, ^*#*^*P* < 0.05 vs. Ang II or 20-HETE group, *n* = 3.

Ang II-induced intracellular Ca^2+^ dysregulation and activation of Ca^2+^-associated signaling pathways are closely linked to cardiac hypertrophy^36^. Ang II and 20-HETE treatments significantly increased intracellular Ca^2+^ concentrations (*P* < 0.05, Fig. 7A). Ca^2+^, as a signaling molecule, activates the Ca^2+^/CaM pathway, triggering the CaN/NFAT signaling cascade and promoting cardiac hypertrophy^37,38^. Ang II and 20-HETE treatments upregulated calcineurin (CaN) protein expression (*P* < 0.05, Fig. 7B) and induced nuclear translocation of cytoplasmic NFAT3 (*P* < 0.05, Fig. 7C). Pretreatment with HET0016 or AAA significantly reduced Ang II-induced increases in intracellular Ca^2+^ levels, CaN expression, and NFAT3 nuclear translocation (*P* < 0.05, Fig. 7A, B and C). These findings suggest that 20-HETE production induced by Ang II activates the CaN/NFAT3 signaling pathway via increased intracellular Ca^2+^ levels, contributing to Ang II-induced cardiac hypertrophy.

## Discussion

**Fig. 8:**
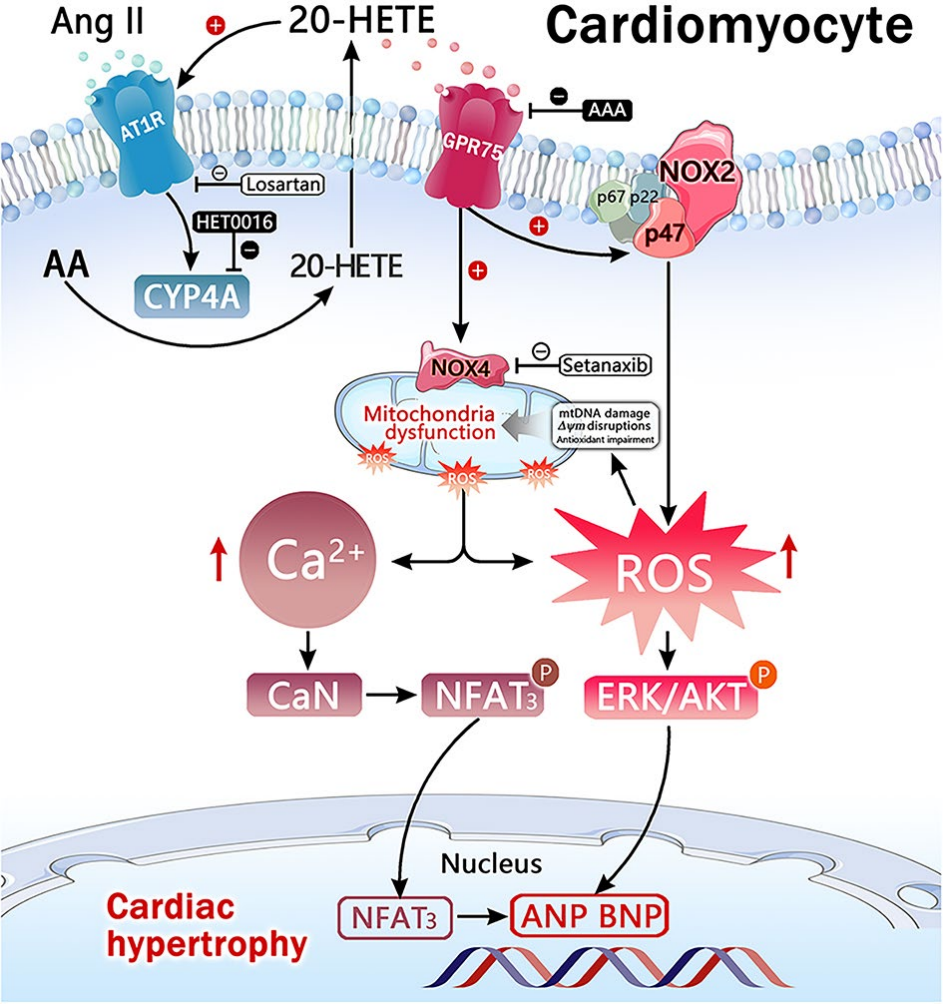
In cardiomyocytes, this study is the first to demonstrate that 20-HETE upregulates AT1 receptor expression, thereby amplifying the biological effects of Ang II. Concurrently, Ang II induces CYP4A expression through an AT1 receptor-mediated signaling pathway, promoting 20-HETE production. Subsequently, 20-HETE activates NOX2 and NOX4 via the GPR75 receptor, leading to excessive intracellular ROS generation and mitochondrial oxidative damage. The 20-HETE/GPR75 axis further mediates Ang II-induced intracellular Ca^2+^ overload, where elevated Ca^2+^ activate calcineurin (CaN) to form the Ca^2+^-CaN complex. This complex promotes NFAT3 dephosphorylation and nuclear translocation, thereby activating hypertrophy-associated gene expression and ultimately leading to cardiac hypertrophy.

This study investigated the mechanisms and molecular pathways underlying the role of 20-HETE in Ang II-induced hypertrophy in H9c2 cells. The results demonstrated that Ang II promoted CYP4A1 expression and 20-HETE production through an AT1 receptor-dependent pathway. Inhibition of 20-HETE synthesis or blockade of its activity effectively suppressed Ang II-induced hypertrophy in H9c2 cells, an effect closely associated with 20-HETE-induced ROS generation, mitochondrial damage, and activation of the Ca^2+^/CaN-NFAT3 signaling pathway. Notably, this research was the first to highlight the significant role of the novel 20-HETE receptor, GPR75, in Ang II-induced cardiac hypertrophy (Fig. 8).

In the vascular system, 20-HETE acts as a potent vasoconstrictor, similar to Ang II, and plays a critical role in blood pressure regulation and the development of CVDs^12,39^. Regarding cardiac function, previous studies demonstrated that under ischemic conditions, such as MIRI, 20-HETE production is upregulated, and its inhibition significantly aids in myocardial recovery post-MIRI^14^. Recent research also identified the involvement of 20-HETE in maladaptive cardiac hypertrophy associated with heart failure. For instance, in a model of ISO-induced cardiac hypertrophy, reducing 20-HETE production through CYP4A inhibition effectively alleviated pathological cardiac remodeling^19^. In this study, 20-HETE exhibited direct hypertrophic effects on H9c2 cells in a dose-dependent manner, leading to increased mRNA expression of ANP and BNP, enhanced total protein synthesis, and enlarged cell surface area.

It has been reported that Ang II activates phospholipase A2 (PLA2) via the AT1 receptor, promoting arachidonic acid release from VSMCs and thereby increasing 20-HETE synthesis^21^. In the kidneys, Ang II upregulates CYP4A expression and 20-HETE production, while reducing 20-HETE synthesis significantly ameliorates Ang II-induced renal fibrosis^40^. Previous research confirmed that 20-HETE mediates Ang II-induced cardiomyocyte apoptosis by promoting ROS generation and mitochondrial damage^25^. Consistent with these findings, we observed that Ang II upregulated CYP4A1 expression and 20-HETE production in H9c2 cells via an AT1 receptor-dependent mechanism. Treatment with HET0016, a specific ω-hydroxylase inhibitor that reduces 20-HETE production, effectively suppressed Ang II-induced cardiomyocyte hypertrophy. Additionally, studies in the vascular system demonstrated that 20-HETE enhances ACE transcription and activity, thus promoting Ang II production^41^. In the kidney, 20-HETE positively regulates the RAAS, synergizing with Ang II to accelerate the progression of hypertension^42^. In the present study, 20-HETE was observed for the first time to promote AT1 receptor expression, further confirming its amplifying effect on the Ang II signaling in cardiomyocytes. These findings suggest that Ang II exerts its effects on the vascular system, kidneys, and heart by stimulating 20-HETE synthesis. Moreover, 20-HETE acts as both a mediator and an amplifier of Ang II’s biological functions by enhancing ACE activity and AT1 receptor expression, thereby creating a positive feedback loop that drives cardiovascular disease progression.

Numerous studies have confirmed that excessive ROS production plays a central role in Ang II-induced cardiac hypertrophy, with many downstream effects of Ang II signaling mediated by ROS and oxidative stress^43,44^. In cardiac tissues, NADPH oxidase is a major source of Ang II-induced ROS^45^. In this study, both Ang II and 20-HETE were shown to significantly increase ROS production in H9c2 cells, while inhibiting 20-HETE synthesis effectively reversed Ang II-induced ROS generation. Pretreatment with the specific NOX1/NOX4 inhibitor Setanaxib significantly reduced 20-HETE-induced ROS production. These results suggest that Ang II promotes 20-HETE synthesis, which activates NADPH oxidase, leading to ROS generation. NADPH oxidase activity was further evaluated, confirming that Ang II and 20-HETE enhanced NADPH oxidase activity, whereas inhibiting 20-HETE synthesis significantly blocked Ang II-induced NADPH oxidase activation.

Among the NOX family enzymes, NOX2 and NOX4 are prominently expressed in cardiomyocytes and cardiac fibroblasts, serving as major ROS sources in the heart^33^. NOX2 activation requires cytoplasmic regulatory subunits such as p47phox and p67phox, with p47phox phosphorylation and membrane translocation being critical^33^. In this study, Ang II and 20-HETE significantly upregulated NOX2 expression and p47phox phosphorylation in H9c2 cells, inducing NADPH oxidase activation and increasing intracellular ROS levels. Unlike NOX2, NOX4 is localized to mitochondria and is closely associated with mt-ROS generation, with its activation regulated at the transcriptional level. A NOX4 overexpression model demonstrated that excessive mt-ROS production exacerbated Ang II-induced cardiac hypertrophy^34^. Another study revealed that targeting mt-ROS with the peptide SS-31 was more effective in mitigating Ang II-induced hypertrophy than enhancing antioxidant enzyme activity using N-acetylcysteine^46^. Here, Ang II and 20-HETE upregulated NOX4 expression and induced mt-ROS overproduction in H9c2 cells, while inhibiting 20-HETE synthesis reversed these effects. Oxidative stress from decreased antioxidant capacity is a key mechanism of cardiac damage^32^. We observed that Ang II-induced reductions in Mn-SOD activity, a critical mitochondrial antioxidant, were reversed by inhibiting 20-HETE synthesis. Furthermore, we found that inhibiting 20-HETE synthesis mitigated Ang II-induced mt-DNA damage, as indicated by reduced 8-OHdG levels, and protected against *ΔΨm* depolarization caused by Ang II. These findings highlight the critical role of 20-HETE in Ang II-induced cardiac hypertrophy through NOX4-dependent mt-ROS production and mitochondrial oxidative stress injury.

Excessive ROS accumulation not only triggers oxidative damage but also acts as a signaling molecule to activate downstream pathways involved in Ang II-induced cardiac hypertrophy. The ROS-sensitive MAPK/ERK and PI3K/Akt pathways are critical in cardiac hypertrophy induced by various factors^35^. In this study, Ang II stimulation significantly increased ERK1/2 and Akt phosphorylation in H9c2 cells, while inhibition of 20-HETE production effectively prevented this phosphorylation. These findings indicate that Ang II-stimulated 20-HETE production in H9c2 cells triggers oxidative stress through NOX-dependent ROS overproduction, leading to mt-DNA damage, mitochondrial dysfunction, and activation of ROS-sensitive ERK/Akt pathways.

Ample evidence links excessive ROS generation in the heart to the activation of Ca^2+^-regulated signaling pathways. Increased ROS generation can directly activate Ca^2+^ channels, such as ryanodine receptors, causing Ca^2+^ leakage from the sarcoplasmic reticulum into the cytoplasm. Additionally, oxidative stress can trigger Ca^2+^ release from mitochondria via permeability transition pores or Na^+^-Ca^2+^ exchangers^47^. It is well established that elevated intracellular Ca^2+^ levels are essential for Ang II-induced cardiac hypertrophy^36^. Previous studies reported that 20-HETE promotes ROS production via a PKC-dependent mechanism, thereby activating L-type Ca^2+^ channels and causing Ca^2+^ overload in cardiomyocytes^16^. Consistently, this study observed significant increases in intracellular Ca^2+^ levels following 20-HETE treatment. Elevated intracellular Ca^2+^ binds to calmodulin (CaM), activating CaN, which in turn binds to NFAT3, induces its dephosphorylation, and facilitates nuclear translocation. This activation drives the transcription of pro-hypertrophic genes, including ANP, BNP, and β-myosin heavy chain (β-MHC)^37,38^. Interestingly, 20-HETE has been shown to induce NFAT3 nuclear translocation in pulmonary VSMCs, suggesting a similar mechanism may contribute to cardiac hypertrophy^48^. For the first time, this study demonstrated that 20-HETE promotes CaN expression and NFAT3 nuclear translocation in H9c2 cells. Importantly, inhibition of 20-HETE synthesis effectively reversed Ang II-induced CaN expression and NFAT3 nuclear translocation, confirming the involvement of 20-HETE in Ang II-induced cardiac hypertrophy via the Ca^2+^/CaN/NFAT3 signaling pathway.

GPR75, recently identified as a receptor mediating the biological actions of 20-HETE, regulates various physiological and pathological processes, including vascular contraction, insulin secretion, and lipid metabolism^49^. The novel antagonist AAA, targeting the 20-HETE/GPR75 axis, has been shown to prevent the development of 20-HETE-dependent CVDs. Agostinucci et al.^50^ demonstrated that AAA treatment reduced hypertension in a transgenic mouse model overexpressing *Cyp4a12*. Similarly, Sedláková et al.^51^ showed that AAA effectively reversed malignant hypertension in the *Cyp1a1-Ren-2* transgenic rat model. Additionally, research by Cárdenas et al.^28^ found that AAA inhibited the malignant transformation of PC-3 prostate cancer cells. In this study, blocking the 20-HETE/GPR75 axis with AAA significantly reversed Ang II-induced cardiac hypertrophy. To our knowledge, this is the first report investigating GPR75’s role in cardiac hypertrophy. Moreover, blocking GPR75 with AAA replicated the effects of 20-HETE inhibition, significantly suppressing Ang II-induced ROS generation and Ca^2+^/CaN/NFAT3 pathway activation, ultimately reversing Ang II-induced cardiac hypertrophy.

## Conclusions

In summary, this study demonstrates that 20-HETE mediates Ang II-induced cardiac hypertrophy and, for the first time, highlights the significant role of the GPR75 receptor in this process. Targeting the reduction of 20-HETE or blocking its receptor action may offer a novel therapeutic approach for CVDs associated with Ang II.

## Electronic supplementary material

Below is the link to the electronic supplementary material.

Supplementary Material 1

## Data Availability

Data supporting the findings of this study are available from the corresponding author upon reasonable request.
