# Supplementary material for: 20-HETE mediates Ang II-induced cardiac hypertrophy via ROS and Ca2+ signaling in H9c2 cells
Source: Sci Rep. 2025 Jan 17;15:2342. doi: 10.1038/s41598-025-85992-2 (PMC11742049; doi:10.1038/s41598-025-85992-2)

Original wsetern blot in the manuscriptmanuscript

Related to Figure. 2D

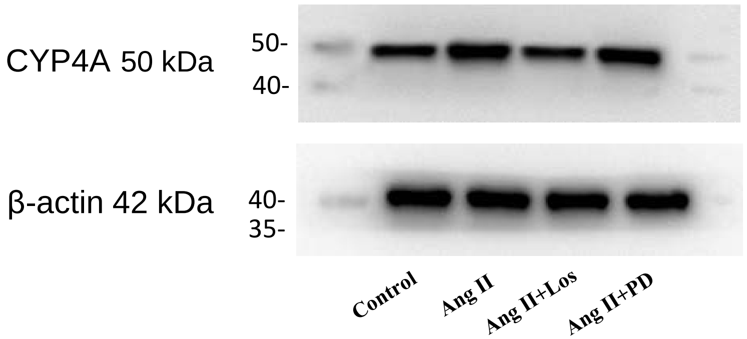

Related to Figure. 2F

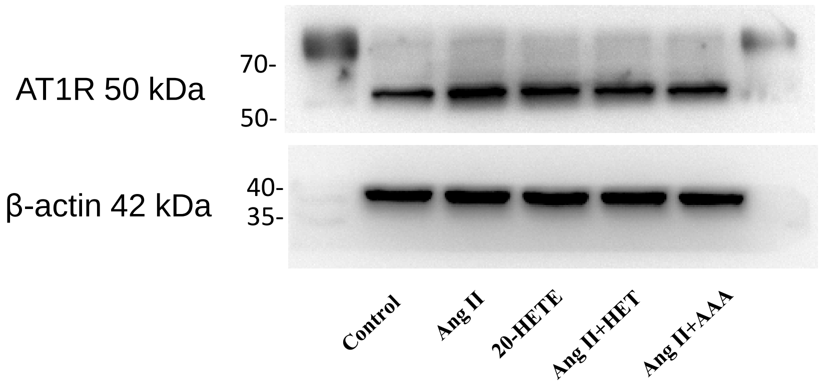

Related to Figure. 3C

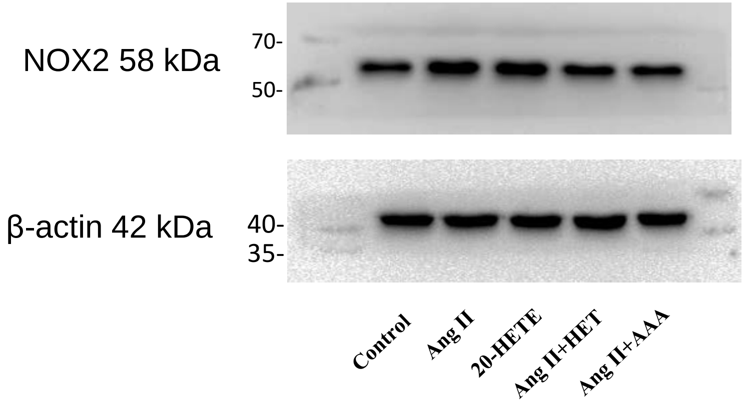

Related to Figure. 3D

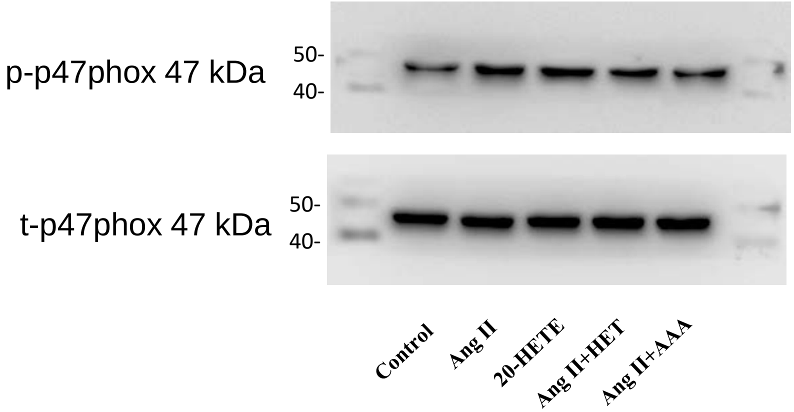

Original wsetern blot in the manuscriptmanuscript

Related to Figure. 4E

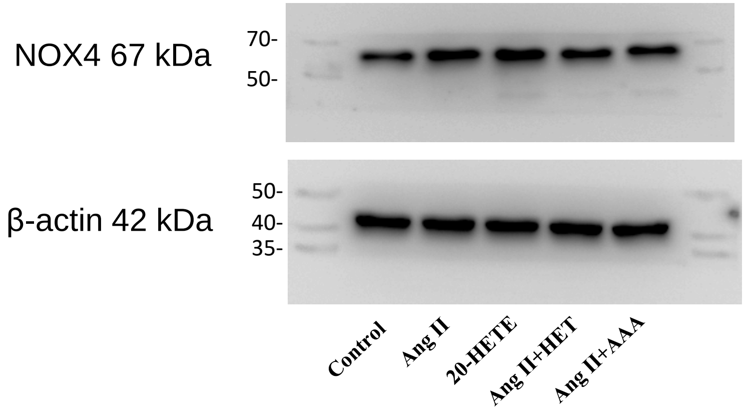

Related to Figure. 6A

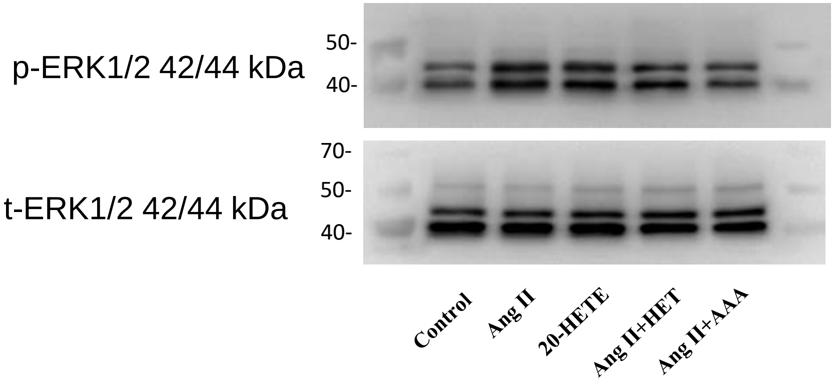

Related to Figure. 6B

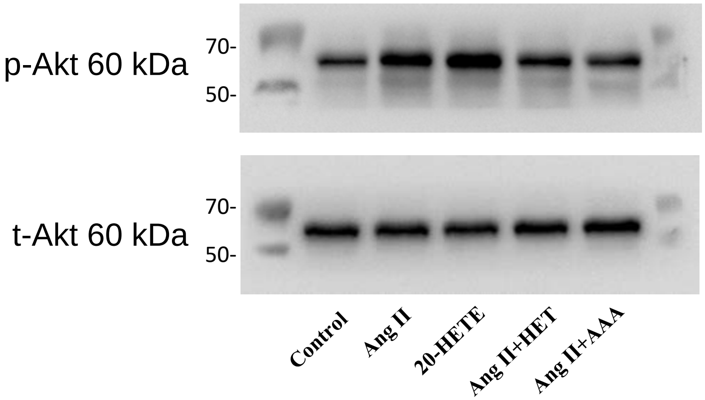

Original wsetern blot in the manuscriptmanuscript

Related to Figure. 7B

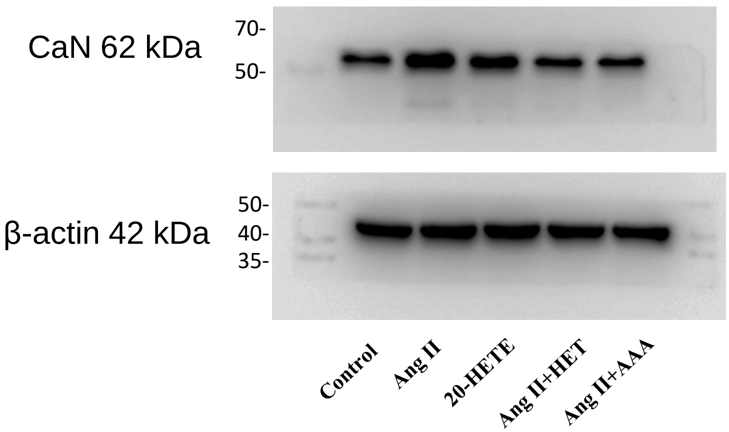

Related to Figure. 7C

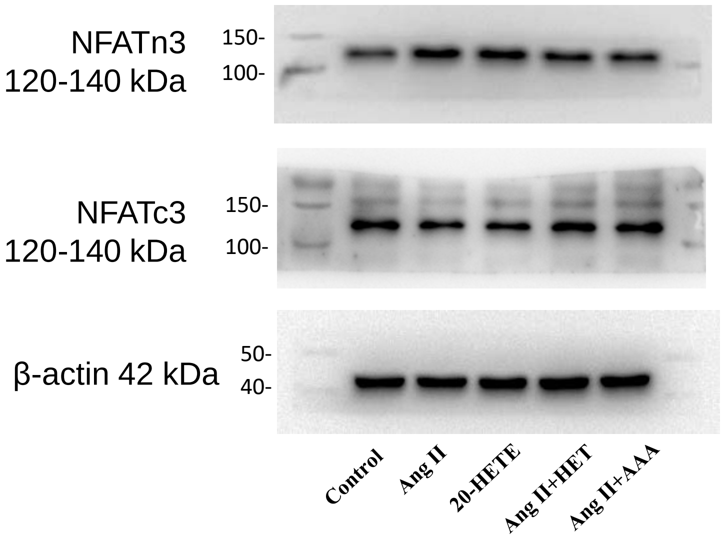

Supplement: Supplementary file 1 — Supplementary Material 1 [file 41598_2025_85992_MOESM1_ESM.pdf]
